# *SLC1A1*, *SLC16A9*, and *CNTN3* Are Potential Biomarkers for the Occurrence of Colorectal Cancer

**DOI:** 10.1155/2020/1204605

**Published:** 2020-05-23

**Authors:** Jie Zhou, Zhiman Xie, Ping Cui, Qisi Su, Yu Zhang, Lijia Luo, Zhuoxin Li, Li Ye, Hao Liang, Jiegang Huang

**Affiliations:** ^1^Guangxi Key Laboratory of AIDS Prevention and Treatment, School of Public Health, Guangxi Medical University, Nanning, Guangxi, China; ^2^The Fourth Hospital of Nanning, Nanning, Guangxi, China; ^3^Guangxi Collaborative Innovation Center for Biomedicine, Life Science Institute, Guangxi Medical University, Nanning, Guangxi, China

## Abstract

**Background:**

This study is aimed at identifying unknown clinically relevant genes involved in colorectal cancer using bioinformatics analysis.

**Methods:**

Original microarray datasets GSE107499 (ulcerative colitis), GSE8671 (colorectal adenoma), and GSE32323 (colorectal cancer) were downloaded from the Gene Expression Omnibus. Common differentially expressed genes were filtered from the three datasets above. Gene Ontology and Kyoto Encyclopedia of Genes and Genomes pathway enrichment analyses were performed, followed by construction of a protein-protein interaction network to identify hub genes. Kaplan-Meier survival analysis and TIMER database analysis were used to screen the genes related to the prognosis and tumour-infiltrating immune cells of colorectal cancer. Receiver operating characteristic curves were used to assess whether the genes could be used as markers for the diagnosis of ulcerative colitis, colorectal adenoma, and colorectal cancer.

**Results:**

A total of 237 differentially expressed genes common to the three datasets were identified, of which 60 were upregulated, 125 were downregulated, and 52 genes that were inconsistently up- and downregulated. Common differentially expressed genes were mainly enriched in the cellular component of extracellular exosome and integral component of membrane categories. Eight hub genes, i.e., *CXCL3*, *CXCL8*, *CEACAM7*, *CNTN3*, *SLC1A1*, *SLC16A9*, *SLC4A4*, and *TIMP1*, were related to the prognosis and tumour-infiltrating immune cells of colorectal cancer, and these genes have diagnostic value for ulcerative colitis, colorectal adenoma, and colorectal cancer.

**Conclusion:**

Three novel genes, *CNTN3*, *SLC1A1*, and *SLC16A9* were shown to have diagnostic value with respect to the occurrence of colorectal cancer and should be verified in future studies.

## 1. Introduction

Colorectal cancer (CRC) is a common malignant tumour of the digestive system. In 2018, 1,800,977 new cases of CRC were identified globally, and the number of deaths attributed to the disease was 861,663 [1]. CRC cells have a strong a strong ability to invade and migrate. Postoperative recurrence and metastasis are the main causes of death in patients with CRC [2]. Although comprehensive treatment measures employed in recent years have improved the five-year survival rate of CRC patients, overall outcomes of treatment remain poor [3].

The occurrence of CRC is closely related to ulcerative colitis (UC) and colorectal adenoma (CRA). Previous studies have shown that repeated stimulation of chronic inflammation is an important factor in the aetiology and pathogenesis of tumours [4, 5]. UC is a nonspecific chronic inflammatory disorder, mainly involving the rectal and colonic mucosa. Typical symptoms include abdominal pain, diarrhoea, purulent stools with blood, and tenesmus. One study found that the risk of CRC in patients with UC is about 10 times higher than that of healthy people. With prolongation of the disease course, the rate of developing CRC in patients with UC over a period of 30 years is about 20% [6]. Furthermore, cancer associated with UC can progress via an inflammation-dysplasia-cancer sequence [7]. Dysplasia, defined as the abnormal development of the neoplastic epithelium that is limited above the basement membrane, is the most reliable hallmark of UC patients with increased risk of malignancy [8]. Dysplasia in UC has two different types of growth patterns, which are either adenoma-like or non-adenoma-like dysplasia-associated lesion or mass (DALM) [9]. Among them, colorectal adenoma-like dysplasia (CRA) has been recognized as precancerous lesions of CRC. In patients with UC, the incidence of CRA can reach 7.5% [10–16]. Moreover, more than 80% of sporadic CRC is transformed from CRA [17–19]. The average time that it takes for CRA with mild atypical hyperplasia to progress to cancer is 18 years, and the average time that it takes from severe atypical hyperplasia is 3.6 years [20]. In short, UC and CRA are important transitional stages in the progression of CRC. With the development of molecular biology technologies, diagnostic markers and gene therapies have the potential to improve the diagnosis and treatment of patients with CRC.

Some gene biomarkers, such as mRNA and miRNAs, have been previously identified to correlate with CRC and developed as diagnostic tools to predict the occurrence, progression, and prognosis of CRC [21–24]. However, the identification of biomarker genes has only been focused on a single stage of CRC in many studies [25–28]. By considering all stages of disease progression, researchers can identify more accurate and targeted diagnostic gene biomarkers to be applied in clinical practice.

In this study, we used bioinformatic methods to identify common differentially expressed genes (DEGs) in UC, CRA, and CRC compared to normal tissues. Gene Ontology (GO) and Kyoto Encyclopedia of Genes and Genomes (KEGG) pathway enrichment analyses were performed, followed by the construction of a protein-protein interaction (PPI) network to screen for hub genes. Kaplan-Meier (KM) survival analysis and TIMER database analysis were used to screen the genes related to the prognosis and tumour-infiltrating immune cells of CRC. Receiver operating characteristic curves (ROC) were used to assess whether the genes could be used as markers for the diagnosis of UC, CRA, and CRC. The results will provide novel diagnostic biomarkers and therapeutic targets for UC, CRA, and CRC at the molecular level and help to develop novel strategies for the prevention and treatment of CRC.

## 2. Materials and Methods

### 2.1. Dataset Sources and Searches

**Figure 1:**
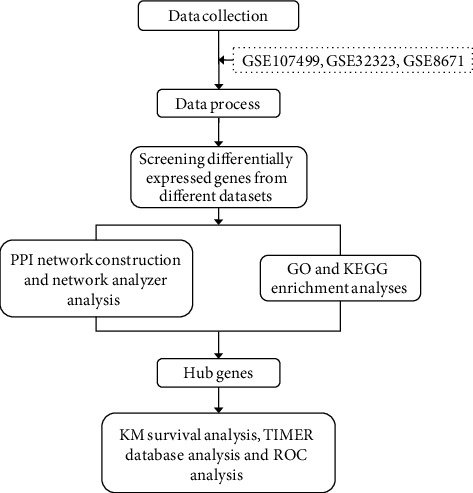
Data analysis pipeline for the identification of clinically relevant genes using microarray datasets.

We conducted a search of Gene Expression Omnibus (GEO) (https://www.ncbi.nlm.nih.gov/geo/), which is a free public functional genomics database including array- and sequence-based data. The search terms ulcerative colitis, colorectal adenomas, and colorectal cancer were used. Datasets were screened according to the following the criteria: (1) samples compared UC/CRA/CRC and normal colorectal tissue, (2) human samples were used, (3) expression profile arrays were performed, (4) raw data were accessible, (5) the number of samples in each group was greater than or equal to five, and (6) the lesion and normal tissue are from the same subject. Reusable datasets for our analysis complied with relevant ethical regulations. The analysis pipeline is shown in Figure 1.

### 2.2. Identification and Integration of Common DEGs

The gene expression profiles were downloaded from the GEO database. Raw data from each dataset were processed using R statistical software (version 3.5.1). The analysis of screened DEGs was carried out using the limma package [29]. The RMA algorithm in the Affy package was used to preprocess data [30]. The classical *t*-test was applied to identify DEGs. The adjusted *p* value < 0.05 and ∣log2FC | >1 were considered cutoff values. Common DEGs from the datasets were integrated by Venn analysis.

### 2.3. GO and KEGG Pathway Enrichment Analyses of Common DEGs

The characteristic biological attributes of common DEGs were identified using GO analysis (http://www.geneontology.org). The functional attributes of commonly identified DEGs were determined using KEGG (http://www.genome.ad.jp/kegg/) pathway enrichment analysis. The Database for Annotation, Visualisation, and Integrated Discovery (DAVID; http://david.abcc.ncifcrf.gov/) [31], a free online tool for the functional classification of genes, was used to conduct GO (biological processes (BP), cellular component (CC), and molecular function (MF)), and KEGG pathway enrichment analyses. A *p* value < 0.05 was set as the cutoff criterion for these analyses.

### 2.4. PPI Network Construction and NetworkAnalyzer Analysis

A PPI network of common DEGs was constructed using the Search Tool for the Retrieval of Interacting Gene (STRING, https://string-db.org/) [32] database. Then, Cytoscape software was utilised to construct a protein interaction relationship network. NetworkAnalyzer software was used to calculate connectivity and identify hub genes. A degree ≥ 5 was set as the cutoff criterion for this analysis.

### 2.5. KM Survival Analysis, TIMER Database Analysis, and ROC Analysis of Hub Genes

Gene-level correlations with patient survival were featured in Gene Expression Profiling Interactive Analysis (GEPIA, http://gepia.cancer-pku.cn/) [33]. The available Cancer Genome Atlas (TCGA) containing patient survival data was used to perform KM survival analysis and to identify CRC-related hub genes. Furthermore, we analysed the correlation of CRC-related hub genes expression with the CRC (colon adenocarcinoma and rectum adenocarcinoma) purity and immune infiltrating levels of B cells, CD4^+^T cells, CD8^+^T cells, neutrophils, macrophages, and dendritic cells by the TIMER database (https://cistrome.shinyapps.io/timer/) [34]. Based on the expression profiles of genes, ROC was performed to assess whether the CRC-related hub genes could be used as markers for the diagnosis of UC, CRA, and CRC.

## 3. Results

### 3.1. Search Results and Differentially Expressed Genes

According to the established inclusion criteria, three datasets, i.e., GSE107499, GSE8671, and GSE32323, were used in our study. Lesions and normal tissue samples were from the same subject. A total of 12 patients with UC were obtained from GSE107499, 32 patients with CRA were obtained from GSE8671, and 17 patients with CRC were obtained from GSE32323. The array datasets in GSE8671 and GSE32323 used the GPL570 platform. GSE107499 used the GPL15207 platform.

**Figure 2:**
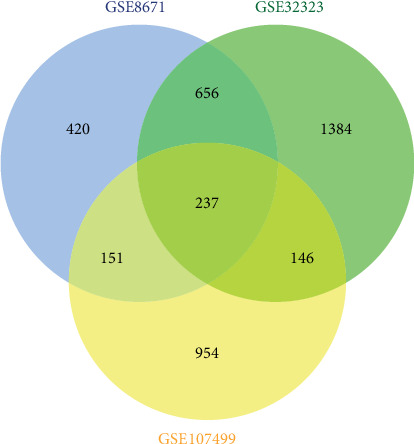
A total of 237 common DEGs were identified by Venn analysis. Differently coloured areas represent different datasets. Overlapping areas signify DEGs shared between datasets.

Expression profile datasets GSE107499, GSE8671, and GSE32323 contained 1488, 1464, and 2423 DEGs that were extracted, respectively. A total of 237 common DEGs were identified using Venn analysis (Figure 2), of which 60 were upregulated and 125 were downregulated; 52 genes were inconsistently up- and downregulated in the three datasets (Table 1). Moreover, 146 common DEGs were identified between UC and CRC, 151 common DEGs were identified between UC and CRA, and 656 common DEGs were identified between CRA and CRC.

### 3.2. GO and KEGG Enrichment Analyses

**Figure 3:**
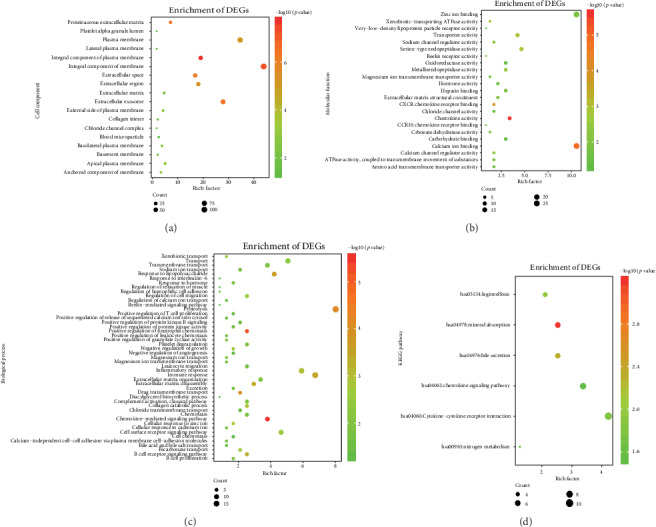
GO enrichment analysis (cellular component (CC, a), molecular function (MF, b), and biological processes (BP, c)) and KEGG pathway analysis (d).

**Figure 4:**
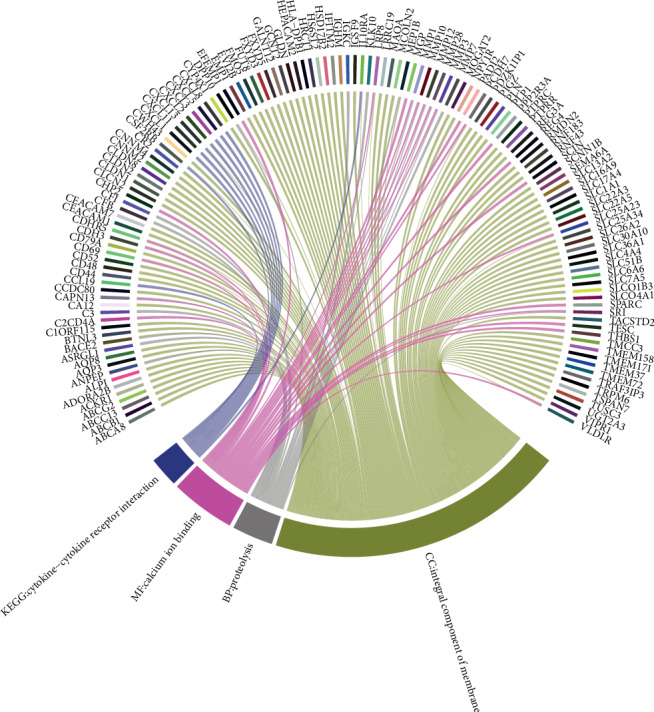
Functional pathways mainly enriched in CC (cellular component), MF (molecular function), and BP (biological processes), and KEGG pathway.

Gene enrichment analysis (Figures 3(a)–3(d)) revealed that common DEGs were markedly enriched in particular CC (Figures 3(a)), which included the extracellular exosome, integral component of membrane, plasma membrane, extracellular space, extracellular region, and integral component of plasma membrane categories (the number of enriched genes was greater than 40). In addition, in BP, genes were mainly enriched in proteolysis. In MF, genes were mainly enriched in calcium ion binding. In the KEGG pathway, genes were mainly enriched in cytokine-cytokine receptor interaction (Figure 4).

### 3.3. PPI Network Analysis

**Figure 5:**
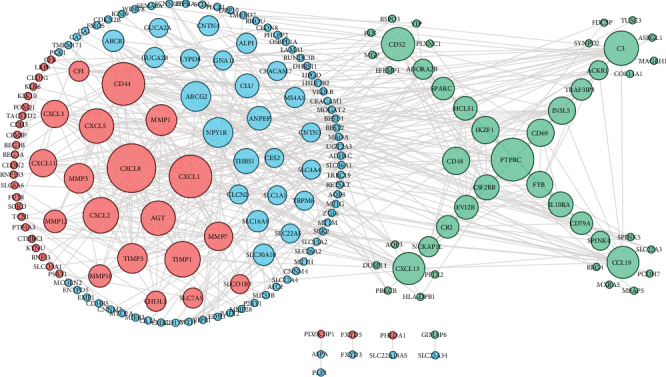
Coexpression network analysis of common DEGs. Red circles represent upregulated genes, blue circles represent downregulated genes, and green circles represent genes that are inconsistently up- and downregulated in the three datasets. The size of each circle corresponds to the degree of differential expression.

The STRING database was used to construct a PPI network with 182 nodes and 455 edges (nodes without connectors were removed) and used the Cytoscape software for visual analysis (Figures 5). Sixty-four hub genes were identified using Cytoscape software. Furthermore, we analysed 42 genes that consistently changed in the three datasets.

### 3.4. KM Survival Analysis, TIMER Database Analysis, and ROC Analysis of Hub Genes

**Figure 6:**
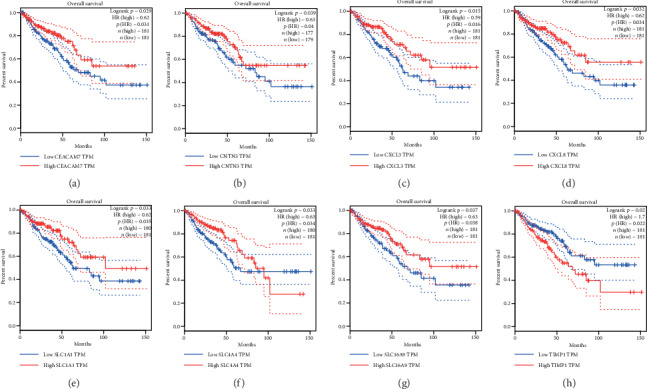
Kaplan-Meier survival analyses of *CEACAM7* (a), *CNTN3* (b), *CXCL3* (c), *CXCL8* (d), *SLC1A1* (e), *SLC4A4* (f), *SLC16A9* (g), and *TIMP1* (h) based on 362 CRC patients (270 colon adenocarcinoma and 92 rectal adenocarcinoma) from the TCGA database.

TCGA database containing 362 CRC patients (270 colon adenocarcinoma and 92 rectum adenocarcinoma) was used for KM survival analyses to screen hub genes related to prognosis of CRC patients. The results showed that the survival rate of CRC patients with high expression of *CEACAM7*, *CNTN3*, *CXCL3*, *CXCL8*, *SLC1A1*, and *SLC16A9* (*p* < 0.05) was higher than that in CRC patients that weakly expressed these genes (Figures 6(a)–6(e) and 6(g)). The trend of *TIMP1* (*p* < 0.05) was the opposite (Figures 6(h)). Those with high expression of *SLC4A4* had a higher survival rate than those with low expression up to near 100 months after the occurrence of CRC, but the opposite trend was seen after 100 months (Figures 6(f)).

**Figure 7:**
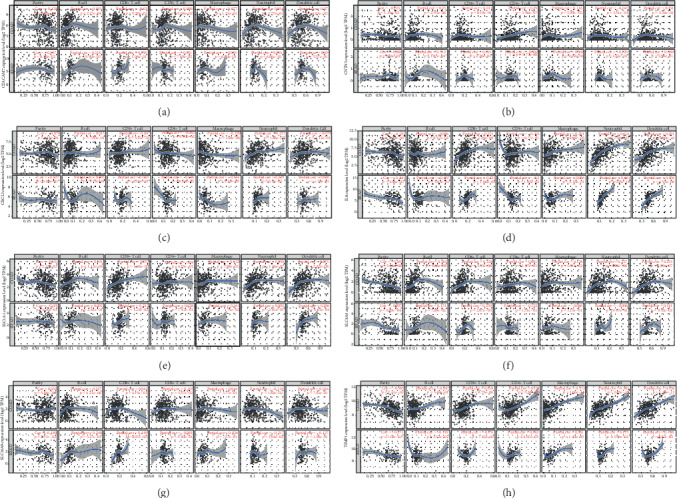
Correlation of *CEACAM7* (a), *CNTN3* (b), *CXCL3* (c), *IL8* (*CXCL8*, d), *SLC1A1* (e), *SLC4A4* (f), *SLC16A9* (g), and *TIMP1* (h) expression with immune infiltration level in COAD (colon adenocarcinoma) patients and READ (rectal adenocarcinoma) patients. The expression level of immune infiltrate markers is represented on the *x*-axis, and the expression level of hub genes is on the *y*-axis. The expression level of immune infiltrate markers and genes are displayed with log2 RSEM.

In colon adenocarcinoma and rectum adenocarcinoma, the expressions of *CEACAM7* and *CNTN3* were significantly positively correlated with infiltrating levels of B cells (Figures 7(a) and 7(b)). The expression of *CXCL3* has significantly negatively related to infiltrating levels of macrophages and had significantly positive correlations with infiltrating levels of neutrophils (Figure 7(c)). The expression of *CXCL8* (*IL8*) was significantly negatively related to CRC purity and had significantly positive correlations with infiltrating levels of CD8^+^T cells, neutrophils, and dendritic cells (Figure 7(d)). The expression of *SLC1A1* was significantly positively correlated with infiltrating levels of CD8^+^T cells and dendritic cells (Figure 7(e)). The expression of *SLC4A4* was significantly negatively related to CRC purity and had significant positive correlations with infiltrating levels of CD8^+^T cells (Figure 7(f)). The expression of *SLC16A9* was significantly negatively related to infiltrating levels of neutrophils and had significantly positive correlations with infiltrating levels of B cells (Figure 7(g)). The expression of *TIMP1* was significantly negatively related to CRC purity and had significantly positive correlations with infiltrating levels of CD4^+^T cells, macrophages, and neutrophils (Figure 7(h)).

**Figure 8:**
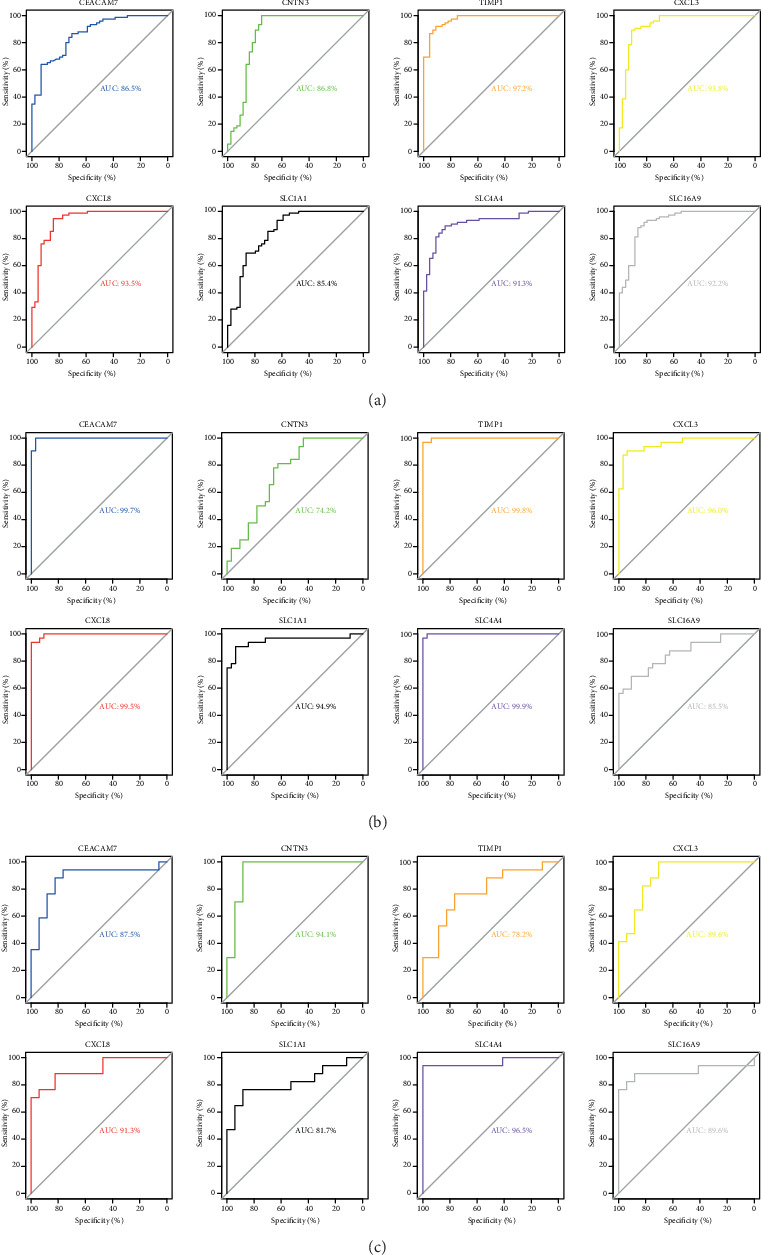
ROC analysis of CRC-related hub genes in the datasets of UC (a), CRA (b), and CRC (c). All *p* < 0.01.

ROC analysis indicated that the area under the curve (AUC) of *CEACAM7*, *CNTN3*, *CXCL3*, *CXCL8*, *SLC1A1*, *SLC4A4*, *SLC16A9*, and *TIMP1* in UC (Figure 8(a)), CRA (Figure 8(b)), and CRC (Figure 8(c)) was greater than 0.7 (*p* < 0.01).

## 4. Discussion

Our study integrated three original microarray datasets, i.e., GSE107499, GSE8671, and GSE32323. The analysis identified 237 common DEGs, including 60 upregulated, 125 downregulated, and 52 genes that were inconsistently up- and downregulated in the three datasets. The Venn analysis suggested that UC-CRA-CRC is a gradual process. Gene enrichment analysis showed that common DEGs were mainly enriched in the cellular component category. Eight hub genes, i.e., *CEACAM7*, *CNTN3*, *CXCL3*, *CXCL8*, *SLC1A1*, *SLC4A4*, *SLC16A9*, and *TIMP1*, were shown to be associated with the prognosis of CRC. These hub genes are related to cancer purity and immune infiltration of different cells in CRC. Specifically, they have strong diagnostic value for UC, CRA, and CRC.

Among the eight hub genes, *CXCL3* (C-X-C motif chemokine ligand 3) and *CXCL8* (C-X-C motif chemokine ligand 8) were upregulated in CRC patients, which is consistent with previous studies [35, 36]. *CXCL3* and *CXCL8* are members of the CXC chemokine family. Studies have shown that chemokines can regulate the proliferation of tumour cells and mediate the infiltration of tumours with immune cells [37, 38]. In our study, we also found that the expression of *CXCL3* in CRC is significantly negatively correlated with the infiltration of macrophages and has significantly positive correlations with the infiltrating levels of neutrophils. Another study confirmed that the *CXCL8* can promote the proliferation and metastasis of a CRC cell line [39]. In addition, *CXCL3* and *CXCL8* are also closely related to UC and CRA. For example, it has been found that *CXCL3* [28] and *CXCL8* [40] participate in the pathogenesis of UC and can be used as therapeutic targets for UC. For CRA, a study by Mclean et al. showed that the inflammatory cytokine genes *CXCL1*, *CXCL2*, *CXCL3*, *CCL20*, *IL8* (*CXCL8*), *CCL23*, *CCL19*, *CCL21*, and *CCL5*are dysregulated in CRA [25]. Hence, our research validates the important role of the *CXCL3* and *CXCL8* in UC, CRA, and CRC.

Multiple studies have also confirmed that *SLC4A4*, *CEACAM7*, and *TIMP1* are related to UC, CRA, and CRC. Bian et al. found that the *SLC4A4* (solute carrier family 4 member 4) and *CEACAM7* (carcinoembryonic antigen-related cell adhesion molecule 7) have been found to be associated with an unfavourable prognosis in CRC [41]. *SLC4A4* was also found to be a differentially expressed gene common to UC and CRC [42]. The downregulation of *CEACAM7* expression in hyperplastic polyps and early adenomas represents some of the earliest observable molecular events leading to CRC [43]. *TIMP1* (tissue inhibitor of metalloproteinase-1) is a member of the tissue inhibitor of metalloproteinase (TIMP) family that inhibits matrix metalloproteinases (MMPs) [44]. TIMP proteins are classically identified as tumour inhibitory based on their capacity to inhibit matrix metalloproteinase- (MMP-) dependent activity [45]. In a number of studies, the expression of *TIMP1* was increased in cancer patients [26, 27, 46]. This phenomenon is also true in our study. In-depth studies have revealed that *TIMP1* accelerates cell proliferation by activating YAP/TAZ in cancer, suggesting that the TIMP1-YAP/TAZ axis may be a novel potential drug target for the treatment of cancer patients [44]. In addition, studies have also shown that *TIMP1* is an important marker of UC [47] and CRA [48].

At present, there is still a lack of research into *SLC1A1*, *SLC16A9*, and *CNTN3* related to intestinal diseases. The downregulated hub gene *SLC1A1* (solute carrier family 1, member 1) is located on chromosome 9p24 and encodes for a member of the high-affinity glutamate aspartate transporter family, which is essential for the transport of glutamate across plasma membranes [49]. The studies related to *SLC1A1* have most commonly involved the investigation of neuropsychiatric disorders [50, 51]. There have been few studies investigating the relationship between *SLC1A1* expression and cancer. Bianchi et al. found that increased expression of *SLC1A1* is correlated with the differentiation of glioma cells [52], and Fan et al. found that *SLC1A1* may play a major role in osteosarcoma development via bioinformatics analysis [53]. Regarding CRC, one study showed that *SLC1A1* expression and glutamate transporter activity were altered in SN38-resistant CRC cells [54]. The downregulated hub gene of *SLC16A9* (solute carrier family 16, member 9), also known as monocarboxylate transporter 9, belongs to a family of proton-linked plasma membrane transporters [55]. The monocarboxylate transporter family now comprises 14 members, of which only the first four have been demonstrated to catalyse the proton-linked transport of metabolically important monocarboxylates such as lactate, pyruvate, and ketone bodies across biological membranes. Malignant tumours rely heavily on aerobic glycolysis and thus need to efflux lactic acid via such transporters to the tumour microenvironment to maintain a robust glycolytic flux and to avoid poisoning themselves [56]. *CNTN3* (Contactin 3) is a member of the contactin family that is primarily expressed in the nervous system. Hence, it may function in the formation and maintenance of specific neuronal networks [57–59]. For cancer, a previous study suggested that *CNTN3* is a potential target gene of hsa-miR-3675b in breast cancer, and it was demonstrated that *CNTN3* may be associated with cell proliferation, apoptosis, and cell cycle progression [60]. Another study shows that the lower expression levels of *CNTN3* may be an independent biomarker that predicts poor overall survival time in patients with glioblastoma multiforme [61]. The above studies indicate that *SLC1A1*, *SLC16A9*, and *CNTN3* are very likely to play important roles in the development of cancer. At the same time, our research also shows that they undergo consistent changes in UC, CRA, and CRC, and all have diagnostic value. Therefore, we speculate that *SLC1A1*, *SLC16A9*, and *CNTN3* are the key factors in the development of CRC.

This study has some limitations. First, biomarkers were only evaluated at the gene level. They require further verification using in vivo experiments. Second, the sample size is not big enough; therefore, statistically significant conclusions cannot be drawn. Third, UC, CRA, and CRC are divided into different subtypes according to different molecular pathways of onset. For example, the chromosomal instability (CIN) pathway and the microsatellite instability (MSI) pathway are the two main molecular pathways leading to CRC. The common differentially expressed genes among CRC, UC, and CRA caused by different pathways may be different. Due to the limitations of the sample source, our study did not conduct further analysis. Moreover, the lack of clinical information for patients included in the microarray datasets could affect the accuracy of the evaluation of the diagnostic value of these biomarkers.

In conclusion, our study identified eight hub genes, i.e. *CXCL3*, *CXCL8*, *CEACAM7*, *CNTN3*, *SLC1A1*, *SLC16A9*, *SLC4A4*, and *TIMP1* by bioinformatics analysis, which have clinical diagnostic value for UC, CRA, and CRC. Among the hub genes, *CXCL3*, *CXCL8*, *CEACAM7*, *CNTN3*, *SLC4A4*, and *TIMP1* have been shown to be related to CRC. Importantly, we found that three novel genes, *SLC1A1*, *SLC16A9*, and *CNTN3* have potential diagnostic value for indicating the occurrence of CRC. It is necessary to further carry out related molecular biological experiments to explore the role of them in CRC progression.

## Figures and Tables

**Table 1:** A total of 237 common DEGs were identified, of which 60 were upregulated, 125 were downregulated, and 52 genes that were inconsistently up- and downregulated in the three datasets.

	Genes
Upregulated genes	*AGT*, *CLDN1*, *FUT8*, *MMP12*, *RNF43*, *AJUBA*, *CLDN2*, *FXYD5*, *MMP3*, *SLC6A6*, *APCDD1*, *CRNDE*, *GRHL1*, *MMP7*, *SLC7A5*, *ARNTL2*, *CTHRC1*, *HS6ST2*, *NEBL*, *SLCO1B3*, *BACE2*, *CXCL1*, *IFITM2*, *OSBPL3*, *SLCO4A1*, *C2CD4A*, *CXCL11*, *KLK10*, *PDZK1IP1*, *SORD*, *CD44*, *CXCL2*, *KLK6*, *PHLDA1*, *TACSTD2*, *CDH3*, *CXCL3*, *KRT6B*, *PSAT1*, *TCN1*, *CEMIP*, *CXCL5*, *KYNU*, *PTP4A3*, *TESC*, *CFB*, *CXCL8*, *LRP8*, *REG1B*, *TIMP1*, *CFI*, *CYP4X1*, *MMP1*, *REG3A*, *TIMP3*, *CHI3L1*, *FOXQ1*, *MMP10*, *RNF183*, and *TMEM158*

Downregulated genes	*A1CF*, *CEACAM7*, *HEPACAM2*, *P2RY1*, *SLC25A23*, *ABCA8*, *CES2*, *HPGD*, *PADI2*, *SLC25A34*, *ABCB1*, *CHP2*, *HRCT1*, *PCK1*, *SLC26A2*, *ABCC13*, *CLCN2*, *HSD17B2*, *PEX26*, *SLC30A10*, *ABCG2*, *CLDN8*, *IGSF9*, *PHLPP2*, *SLC36A1*, *ADH1C*, *CLU*, *ISX*, *PIGZ*, *SLC4A4*, *ALPI*, *CMBL*, *ITPKA*, *PKIB*, *SLC51B*, *ANPEP*, *CNNM2*, *LAMA1*, *PLCE1*, *SRI*, *APPL2*, *CNNM4*, *LRRC19*, *PLP1*, *TEX11*, *AQP8*, *CNTN3*, *LYPD8*, *PPP2R3A*, *THBS1*, *ASPA*, *CNTN4*, *MAOA*, *PTPRR*, *THRB*, *BEST2*, *CWH43*, *MCOLN2*, *RETSAT*, *TMCC3*, *BEST4*, *DHRS11*, *MEP1B*, *RHOU*, *TMEM171*, *BTNL3*, *DPP10*, *MIER3*, *RMDN2*, *TMEM37*, *C1orf115*, *EDN3*, *MMP28*, *RUNDC3B*, *TMEM72*, *C2orf88*, *EMP1*, *MOGAT2*, *SCIN*, *TRIM36*, *CA1*, *ENTPD5*, *MS4A1*, *SCNN1B*, *TRPM6*, *CA12*, *FMO5*, *MT1F*, *SEMA6A*, *TSPAN7*, *CA7*, *FXYD3*, *MT1G*, *SGK2*, *TUBAL3*, *CAPN13*, *GALNT12*, *MT1H*, *SLC13A2*, *UGT2A3*, *CD177*, *GBA3*, *MT1M*, *SLC16A9*, *USP2*, *CDHR5*, *GCNT2*, *MYO1A*, *SLC17A4*, *VIPR1*, *CDKN2B*, *GNA11*, *NPY1R*, *SLC1A1*, *VLDLR*, *CDKN2B-AS1*, *GUCA2A*, *NXPE4*, *SLC22A18AS*, *WDR78*, *CEACAM1*, *GUCA2B*, *OSBPL1A*, *SLC22A5*, and *ZG16*

The genes that are inconsistently up- and downregulated in the three datasets	*ACKR1*, *CD79A*, *FYB*, *MAGEH1*, *PTPRC*, *TUSC3*, *ADORA2B*, *COL14A1*, *GIMAP6*, *MFAP5*, *RASSF2*, *VIP*, *AQP3*, *CR2*, *GLDN*, *MGP*, *REG4*, *ASRGL1*, *CSF2RB*, *HCLS1*, *MXRA5*, *RSPO3*, *C3*, *CXCL13*, *HLA-DPB1*, *NCKAP1L*, *SLC22A3*, *CCDC80*, *DUSP14*, *IGHM*, *PCDH7*, *SPARC*, *CCL19*, *EFEMP1*, *IGKC*, *PITX2*, *SPINK4*, *CD48*, *EVI2B*, *IKZF1*, *PLN*, *SPINK5*, *CD52*, *FAM129A*, *IL10RA*, *PLXNC1*, *SYNPO2*, *CD69*, *FDCSP*, *INSL5*, *PRKCB*, and *TRAF3IP3*

## Data Availability

The datasets of GSE107499, GSE8671, and GSE32323 can be obtained from Gene Expression Omnibus.
